# All coronary arteries originating from the right sinus of Valsalva: a multimodality imaging approach

**DOI:** 10.1097/MCA.0000000000001505

**Published:** 2025-01-20

**Authors:** Alexander Suchodolski, Aleksandra Korus, Dariusz Kucias, Jan Głowacki, Mariola Szulik

**Affiliations:** a Doctoral School of the Medical University of Silesia in Katowice; b Department of Cardiology and Electrotherapy, Silesian Center for Heart Diseases; c Student Research Group; d Department of Radiology and Radiodiagnostics, Faculty of Medical Sciences in Zabrze, Medical University of Silesia, Katowice; e Computed Tomography Laboratory, Silesian Centre for Heart Diseases, Zabrze; f Department of Medical and Health Sciences, Collegium Medicum – Faculty of Medicine, Faculty of Applied Sciences, WSB University, Dąbrowa Górnicza, Poland

A 59-year-old male patient presented with fatigue and lower limb swelling. His medical history included well-controlled arterial hypertension, atrial flutter, heart failure, and a previously implanted cardioverter-defibrillator.

Coronary angiography was performed, but due to suspicion of a coronary anomaly, computed tomography (CT) was subsequently carried out. The CT revealed that all coronary arteries arose from the right coronary sinus (Fig. 1) (Supplementary Figure 1, Supplemental digital content 1, http://links.lww.com/MCA/A747).

**Fig. 1:**
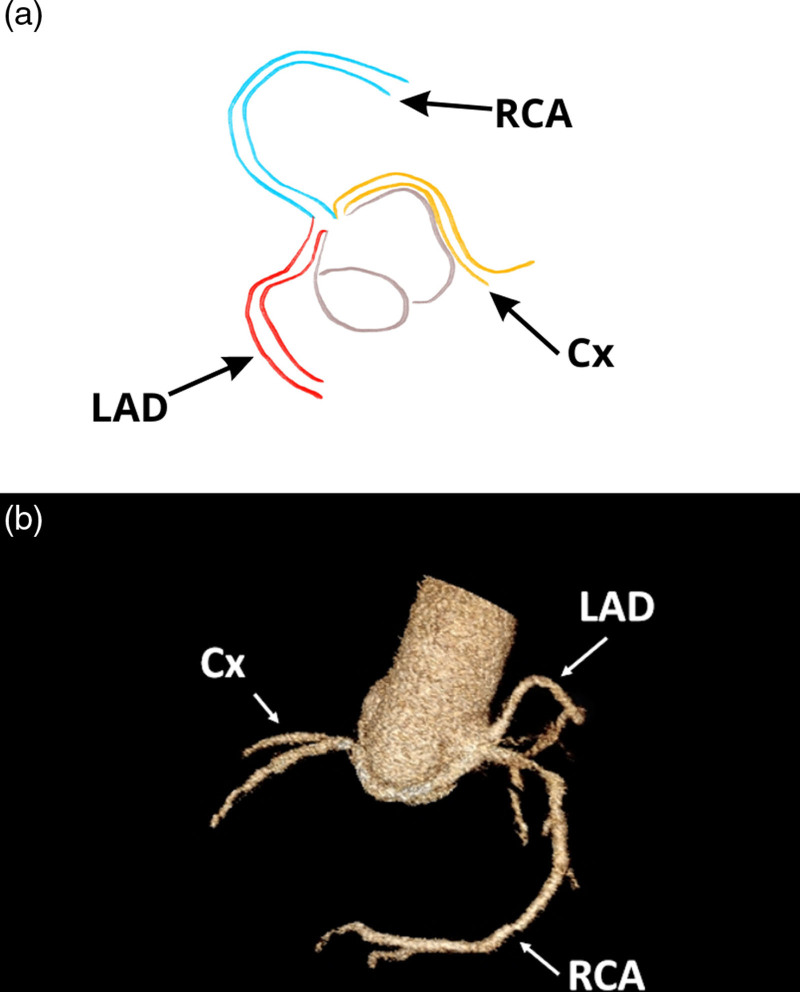
An illustration of the top view of the heart with the schematic presentation of the patient’s coronary anatomy (a). Volume rendering technique reconstructions of computed tomography images showing the origins of all coronary arteries from the right sinus of Valsalva (b). Cx, circumflex artery; LAD, left anterior descending artery; RCA, right coronary artery.

The left anterior descending artery originated from the proximal part of the right coronary artery and coursed between the pulmonary trunk and the sternum. The circumflex artery and its diagonal branch arose directly from the right coronary sinus, initially running between the aorta and right ventricular outflow tract before continuing intramuscularly.

Follow-up echocardiography revealed a positive retroaortic anomalous coronary (RAC) sign. The left ventricular ejection fraction was 25% with significant hypokinesis of the lateral wall (Supplementary Figure 2, Supplemental digital content 2, http://links.lww.com/MCA/A748). No other pathologies were found (Fig. 2).

**Fig. 2:**
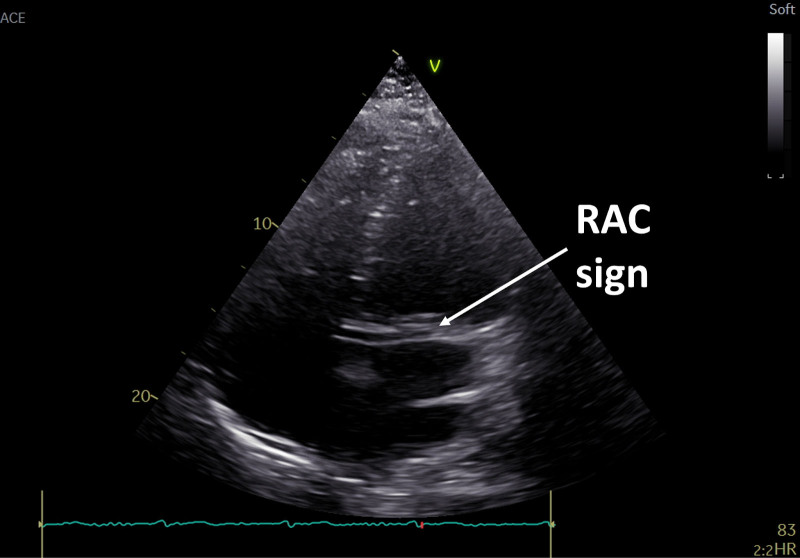
Transthoracic echocardiography images showing the retroaortic anomalous coronary (RAC) sign in a modified four-chamber view.

The patient’s medical management remained unchanged following these findings.

This case presents an extremely rare coronary anatomy [1]. The presence of the RAC sign suggests an anomalous coronary artery with a retroaortic course, but this case demonstrates that multiple anomalies can coexist. RAC sign has a sensitivity of 63.3% and a specificity of 93.3% [2].

Cardiac CT angiography is recommended for a more comprehensive evaluation of such vasculature as it allows for a more comprehensive assessment of heart anatomy than coronary angiography, as shown in this case.

## Acknowledgements

### Conflicts of interest

There are no conflicts of interest.

## Supplementary Material
