# Supplementary figures and images for: All coronary arteries originating from the right sinus of Valsalva: a multimodality imaging approach
Source: Coron Artery Dis. 2025 Jan 20;36(5):453–4. doi: 10.1097/MCA.0000000000001505 (PMC12199794; doi:10.1097/MCA.0000000000001505)

Supplement Figure 1:

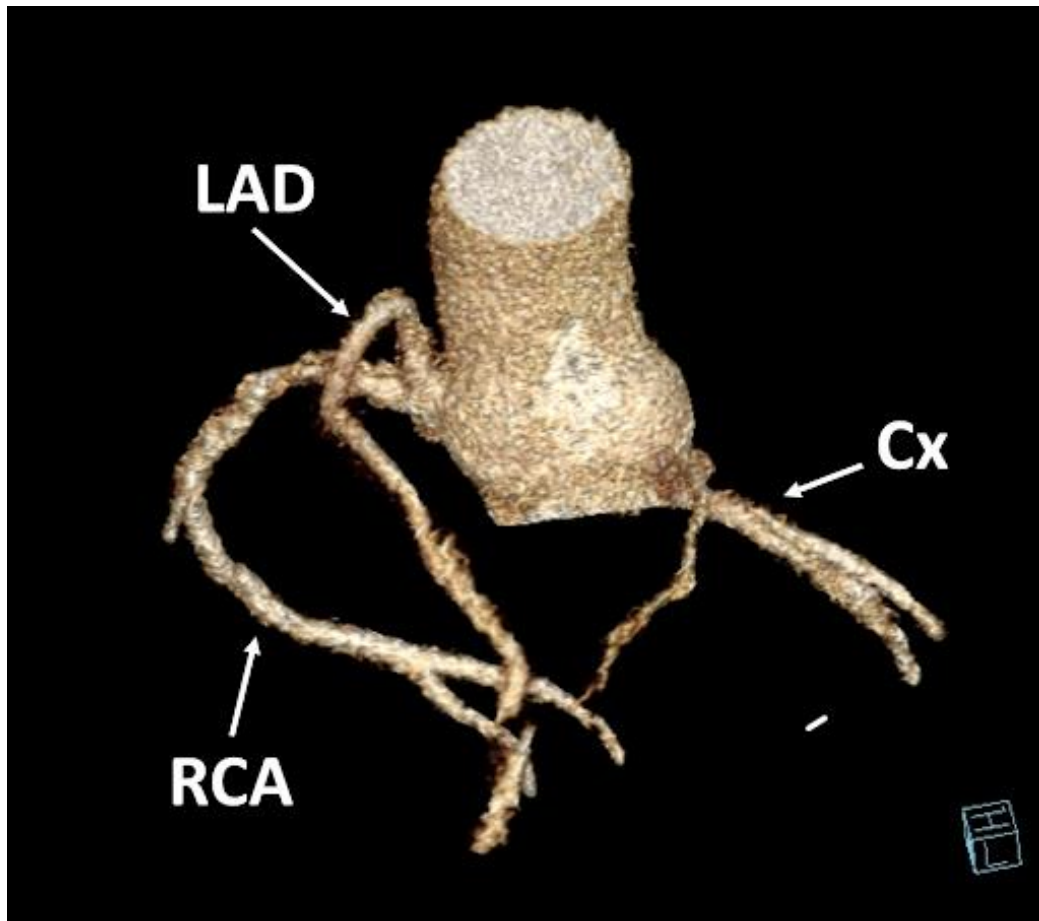

Supplement: Supplementary file 1 [file cad-36-453-s001.pdf]

Supplement Figure 2:

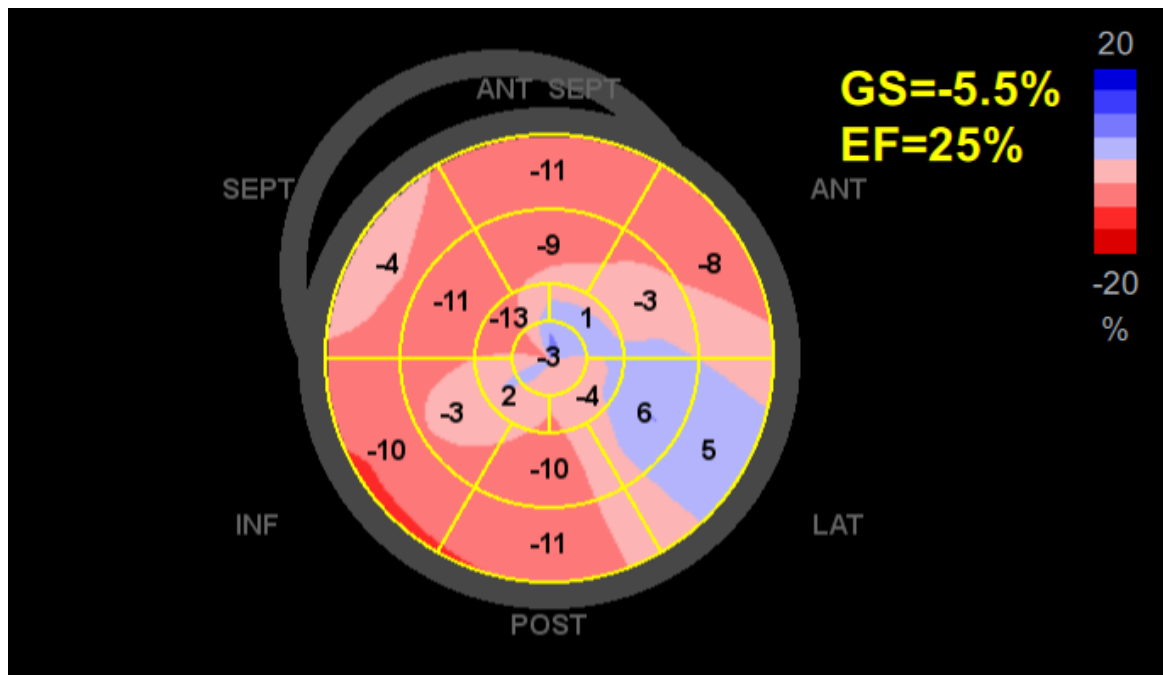

Supplement: Supplementary file 2 [file cad-36-453-s002.pdf]
